# Cannabidiol as a Therapeutic Target: Evidence of its Neuroprotective and Neuromodulatory Function in Parkinson’s Disease

**DOI:** 10.3389/fphar.2020.595635

**Published:** 2020-12-15

**Authors:** Felipe Patricio, Alan Axel Morales-Andrade, Aleidy Patricio-Martínez, Ilhuicamina Daniel Limón

**Affiliations:** ^1^ Laboratorio De Neurofarmacología, Facultad De Ciencias Químicas, Benemérita Universidad Autónoma de Puebla, Puebla, Mexico; ^2^ Facultad De Ciencias Biológicas, Benemérita Universidad Autónoma de Puebla, Puebla, Mexico

**Keywords:** cannabidiol (CBD), neuroprotective, neuromodulatory, l-DOPA-induced dyskinesia, parkinson’s diasese

## Abstract

The phytocannabinoids of *Cannabis sativa* L. have, since ancient times, been proposed as a pharmacological alternative for treating various central nervous system (CNS) disorders. Interestingly, cannabinoid receptors (CBRs) are highly expressed in the basal ganglia (BG) circuit of both animals and humans. The BG are subcortical structures that regulate the initiation, execution, and orientation of movement. CBRs regulate dopaminergic transmission in the *nigro-striatal* pathway and, thus, the BG circuit also. The functioning of the BG is affected in pathologies related to movement disorders, especially those occurring in Parkinson’s disease (PD), which produces motor and non-motor symptoms that involving GABAergic, glutamatergic, and dopaminergic neural networks. To date, the most effective medication for PD is levodopa (l-DOPA); however, long-term levodopa treatment causes a type of long-term dyskinesias, l-DOPA-induced dyskinesias (LIDs). With neuromodulation offering a novel treatment strategy for PD patients, research has focused on the endocannabinoid system (ECS), as it participates in the physiological neuromodulation of the BG in order to control movement. CBRs have been shown to inhibit neurotransmitter release, while endocannabinoids (eCBs) play a key role in the synaptic regulation of the BG. In the past decade, cannabidiol (CBD), a non-psychotropic phytocannabinoid, has been shown to have compensatory effects both on the ECS and as a neuromodulator and neuroprotector in models such as 6-hydroxydopamine (6-OHDA), 1-methyl-4-phenyl-1,2,3,6-tetrahydropyridine (MPTP), and reserpine, as well as other PD models. Although the CBD-induced neuroprotection observed in animal models of PD has been attributed to the activation of the CB1 receptor, recent research conducted at a molecular level has proposed that CBD is capable of activating other receptors, such as CB2 and the TRPV-1 receptor, both of which are expressed in the dopaminergic neurons of the *nigro-striatal* pathway. These findings open new lines of scientific inquiry into the effects of CBD at the level of neural communication. Cannabidiol activates the PPARγ, GPR55, GPR3, GPR6, GPR12, and GPR18 receptors, causing a variety of biochemical, molecular, and behavioral effects due to the broad range of receptors it activates in the CNS. Given the low number of pharmacological treatment alternatives for PD currently available, the search for molecules with the therapeutic potential to improve neuronal communication is crucial. Therefore, the investigation of CBD and the mechanisms involved in its function is required in order to ascertain whether receptor activation could be a treatment alternative for both PD and LID.

## Cannabidiol: Origin, Pharmacokinetics, and Pharmacodynamics

**FIGURE 1:**
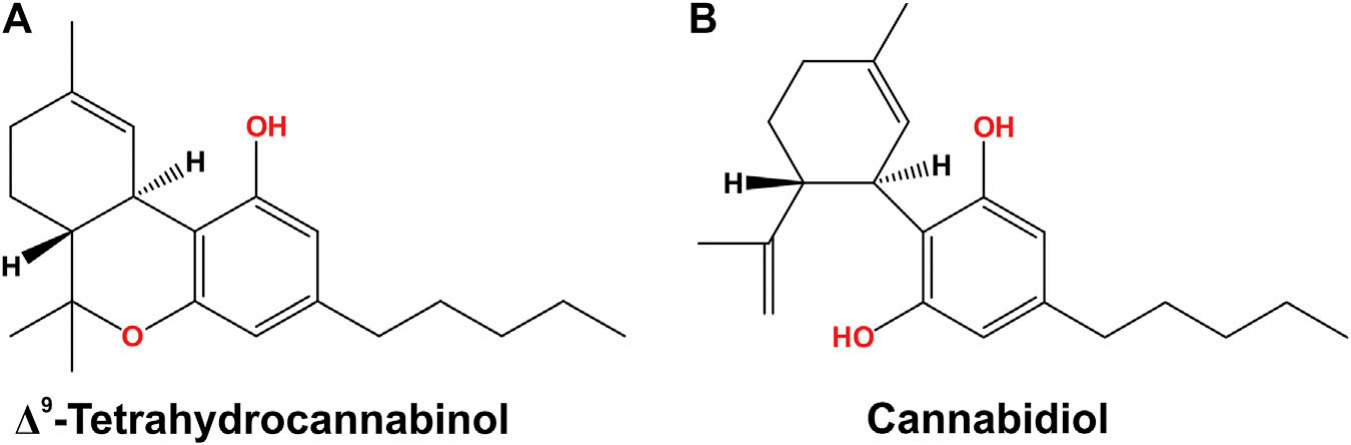
Chemical structures, **(A)** THC and **(B)** CBD, the main phytocannabinoids extracted from the Cannabis plant THC, tetrahydrocannabinol; CBD, cannabidiol.

Taxonomically, *Cannabis sativa* L. pertains to the Cannabaceae family (Russo, 2007), which has recently been found to include the genera *Cannabis*, *Humulus*, and *Celtis*. *Cannabis sativa* has three varieties, *sativa*, *indica* and *ruderalis* (McPartland, 2018). Phytocannabinoids are the active compounds in *Cannabis sativa* L., the most abundant compound of which is Δ^9^-tetrahydrocannabidiol (THC), which has psychoactive pharmacological effects, while cannabidiol (CBD), its second most abundant compound, has psychoactive/non-psychotropic pharmacological effects and is more medically promising than THC (Ibeas Bih et al., 2015) (Figure 1). THC and CBD are initially formed as carboxylic acids (e.g., Δ^9^- THCA, CBDA) that are decarboxylated into neutral form, a process occurring naturally as the plant ages and when it is exposed to light or heat (Hanuš et al., 2016; Wang et al., 2016).

While preclinical and clinical studies have shown that THC induces anxiety and psychotic symptoms in healthy subjects, the consumption of CBD has been found to significantly reduce the effects of THC, with CBD shown to have an antagonistic effect against THC (Dalton et al., 1976; Zuardi et al., 2006). Interestingly, both compounds have been shown to affect inflammation, anxiety, emesis, and nausea; moreover, it has been proposed that they act as both neuroprotective agents and antioxidants (Pertwee 2004; Cascio and Pertwee, 2014). The strategic use of both compounds has been reported for pain relief in cancer and neuropathic pain relief in multiple sclerosis (Zajicek and Apostu, 2011; Fine and Rosenfeld, 2014; Dariš et al., 2019). Studies on CBD have shown that it participates in the regulation of the endocannabinoid system (ECS), the important characteristics of which will be summarized in this review.

**FIGURE 2:**
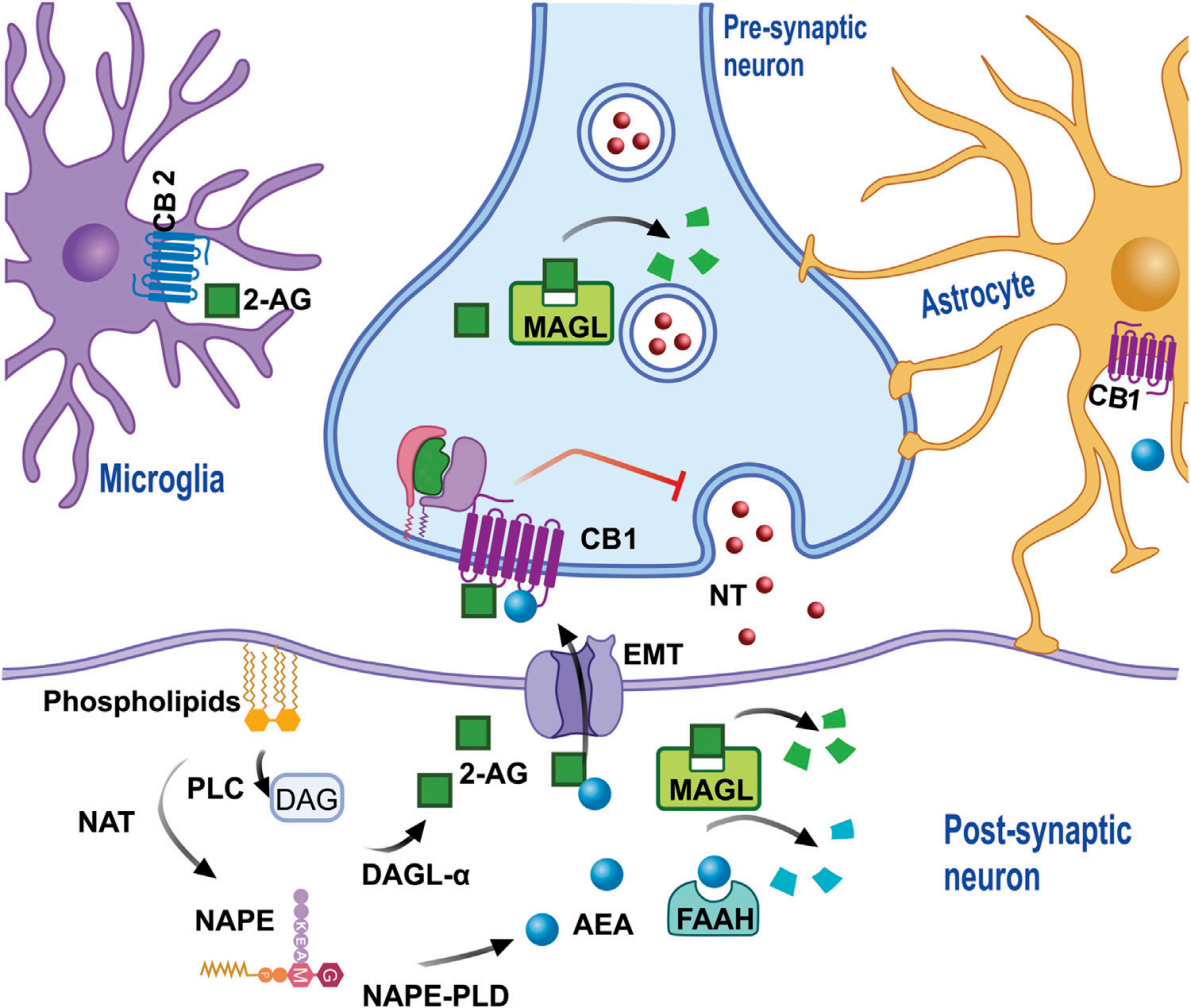
eCB is synthesized from membrane phospholipids. NAT synthesizes the precursor NAPE, which subsequently, through the action of PLD, produces AEA in the cytoplasm of the post-synaptic neuron (or neuron spine). AEA leaves the cytoplasm and enters the synaptic space via diffusion and/or the action of EMT in order that, once it has onside, AEA activates the cannabinoid receptors which inhibit the release of NT. The degradation of AEA in EMT is regulated by FAAH, which produces metabolites such as AA and ETA. 2-AG requires the formation of the DAG precursor by PLC, which then through the action of diacylglycerol lipase α, and together with arachidonic acid generates 2-AG, which then leaves the synaptic space to activate cannabinoid receptors, which are also present in the microglia and/or astrocytes, and can be degraded by MAGL both in the pre and post-synapse, generating AA and Gro as metabolites. Abbreviations: eCB, endocannabinoids; NAT, N-acyl transferase; NAPE, N-acyl-phosphatidylethanolamine; PLD, phospholipase D; AEA, anandamide; NT, neurotransmitters; EMT, endocannabinoid membrane transporter; FAAH, fatty acid amide hydrolase; AA, arachidonic acid; ETA, ethanolamine; 2-AG, 2- Arachidonoylglycerol; DAG, diacylglycerol; PLC, phospholipase C; MAGL, monoacylglycerol lipase.

The ECS is a complex lipid network consisting of cannabinoid receptors (CBRs), endogenous ligands, and the enzymes involved in endocannabinoid degradation and synthesis (Figure 2). Chemicals derived from fatty acid amides and diacylglycerols, endocannabinoids (eCBs) are synthesized endogenously in mammals and are produced on demand in response to increased intracellular calcium levels ([Ca^2+^]_i_) (Di Marzo et al., 1998; Mechoulam and Parker, 2013). The main eCBs are arachidonoylethanolamine, also known as anandamide (AEA), and 2-arachidonoyl-glycerol (2-AG) (Devane et al., 1992; Mechoulam et al., 1995; Sugiura et al., 1995).

The synthesis of AEA is produced by the hydrolysis of a phospholipid precursor, N-acyl-phosphatidylethanolamine (NAPE), which is carried out by the enzyme N-acyl-phosphatidylethanolamine-phospholipase D (NAPE-PLD). There is evidence that AEA is formed from N-acyl-lysophosphatidylethanolamine (NALPE) by the enzymes lysophospholipase D (lysoPLD), α/β-hydrolase 4 (ABH4), and phospholipase C (PLC). The other eCB, 2-AG, is synthetized via the activation of a PLC, thus producing 1,2-diacylglycerol (DAG), which, in turn, is converted into 2-AG by diacylglycerol lipase (DAGL), which can also be synthesized from sn-1-lysophospholipids, via the sequential action of phospholipase A1 (PLA1) and lysophospholipase C (Di Marzo et al., 2015). The main degradation enzymes of the eCBs are fatty acid amide hydrolase (FAAH) and monoacylglycerol lipase (MAGL). FAAH is located in both the Soma and the post-synaptic neuronal dendrites and is associated with the membranes of cytoplasmic organelles that serve as a reservoir of [Ca^2+^]_i_, the mitochondria, and the smooth endoplasmic reticulum. DAGL and MAGL are located in the postsynaptic dendrites and the pre-synaptic neurons, respectively, whit the latter expressed when 2-AG, the main substrate, is metabolized. Both AEA and 2-AG are metabolized by FAAH, with other enzymes, such as the α/β-hydrolase families 6 and 12 (ABHD6 and ABHD12), also participating, although to a lesser extent (<10%) (Mechoulam and Parker, 2013).

Unlike the classical form of neurotransmitter release, eCBs are released from the post-synaptic neuron to then interact with its specific receptors in a retrograde manner (Di Marzo et al., 1998; Di Marzo et al., 2015). It has been proposed that the release of eCBs, but mainly AEA, occurs via a transporter called the *endocannabinoid membrane transporter*: (Yates and Barker, 2009; Fowler 2013). Once released into the synaptic space, these eCBs interact with their specific receptors (Figure 2). CBRs have been cloned, characterized, and classified into two subtypes, cannabinoid receptor type 1 (CB1) (Matsuda et al., 1990) and cannabinoid receptor type 2 (CB2) (Munro et al., 1993), which are proteins containing seven transmembrane domains coupled to inhibitory G proteins (G*α*_i_). At a molecular level, CB1-receptor activation inhibits the release of presynaptic neurotransmitters via the inhibition of the enzyme adenylyl cyclase (AC), the adenylate monophosphate circle/protein kinase A (cAMP/PKA) pathway, and the inhibition of the voltage-dependent Ca^2+^ channels (Mackie, 2006; Kano et al., 2009). This physiological mechanism ensures that the ECS plays a neuromodulatory role.

The pharmacokinetics of CBD is variable and depends on the route of administration (oral, intravenous, sublingual, topical, inhalation, and transdermal), the type of product administered, concomitant food intake, and drug-drug interactions. Pharmaceutical forms with lipid excipients have been reported to improve CBD absorption (Zgair et al., 2016), with a study, conducted on subjects who had ingested food prior to administration via an erosol containing THC/CBD, finding a five-fold increase in the area under the curve (AUC) and a three-fold increase in the C*max*, as well as the prolongation of the T*max* (Stott et al., 2013). The use of sublingual drops at a dose of 20 mg obtained a T*max* of 2.17 h and a C*max* of 2.05 ng/ml (Guy and Flint, 2004). Administration via inhalation obtained a T*max* of 0.17 h and a C*max* of 28.2 ng/ml in occasional users, while a T*max* of 0.29 h and a C*max* of 76.3 ng/ml were obtained in frequent users, both via the administration 1.5 mg doses (Swortwood et al., 2017). Idiosyncratic differences mean that the mechanisms of administering cannabinoids are highly variable, with the oral administration of CBD shown to have a bioavailability of 13–19% (Mechoulam et al., 2002). Vaporization should be considered a promising route of administration, given that it can improve bioavailability without presenting a risk to the consumer, as long as it is used correctly (Varlet et al., 2016).

Cannabidiol is mainly excreted in feces, with part of the drug excreted unchanged (Perez-Reyes et al., 1976), while approximately 100 CBD metabolites are estimated to be excreted via the kidneys (Huestis, 2007). It is mainly metabolized via both oxidation and hydroxylation at various sites in the molecule, generating a complex degradation process. Other metabolites are formed via the *ß*-oxidation and biotransformation of the pentyl side chain and hydroxylations at C-6 and C-7 (Harvey and Mechoulam, 1990), while the highest concentration metabolites are 7-COOH-CBD and 6- OH-CBD, which are excreted intact or as glucuronide acid conjugates (Devinsky et al., 2018).

The metabolite 7-OH-CBD has been reported to both inhibit FAAH (IC50 = 34 μM) and decrease the metabolism of AEA in a basophil culture (IC50 = 50 μM) (Bisogno et al., 2001). Moreover, both 7-OH-CBD and 7-COOH-CBD have been reported to have anti-inflammatory effects and inhibit the production of nitric oxide (NO), reactive oxygen species (ROS), and tumor necrosis factor-alpha (TNF-α) (Mechoulam et al., 2010).

While the inhibition of the CYP1A1, 1A2, 2C9, and 1B1 isoforms has been reported, the inhibition of the CYP2C19 and 3A4 isoforms is more potent (Bornheim and Grillo, 1998; Zendulka et al., 2016; Arellano et al., 2017). This inhibition can have a synergistic effect in the presence of barbiturates (Paton and Pertwee 1972). Although CBD can interrupt the hydrolysis of THC by Cytochrome P450, no pharmacokinetic changes are reported in either compound. CBD metabolites, such as 7-OH or 7-COOH-CBD, have anti-inflammatory effects and inhibit the formation of NO, ROS, TNF-α, IL-1ß, NF-κβ, and IL-6 (Watzl et al., 1991; Kozela et al., 2010; Mechoulam et al., 2010). In addition, CBD reduces the production of prostaglandins (Costa et al., 2004) and nitric oxide synthases (NOS) (Esposito et al., 2006), indicating a route for anti-inflammatory or antinociceptive effects, while one effect of 6-oxo-CBD (a CBD metabolite) is anticonvulsant activity (Carlini et al., 1975). As CBD does not affect the metabolism of 2-AG (Rimmerman et al., 2011), it does not influence the action of the CB1 and CB2 receptors. It should be noted that the effects of eCB and phytocannabinoids, especially CBD, depend on the expression and anatomical location of CBRs in the brain.

**FIGURE 3:**
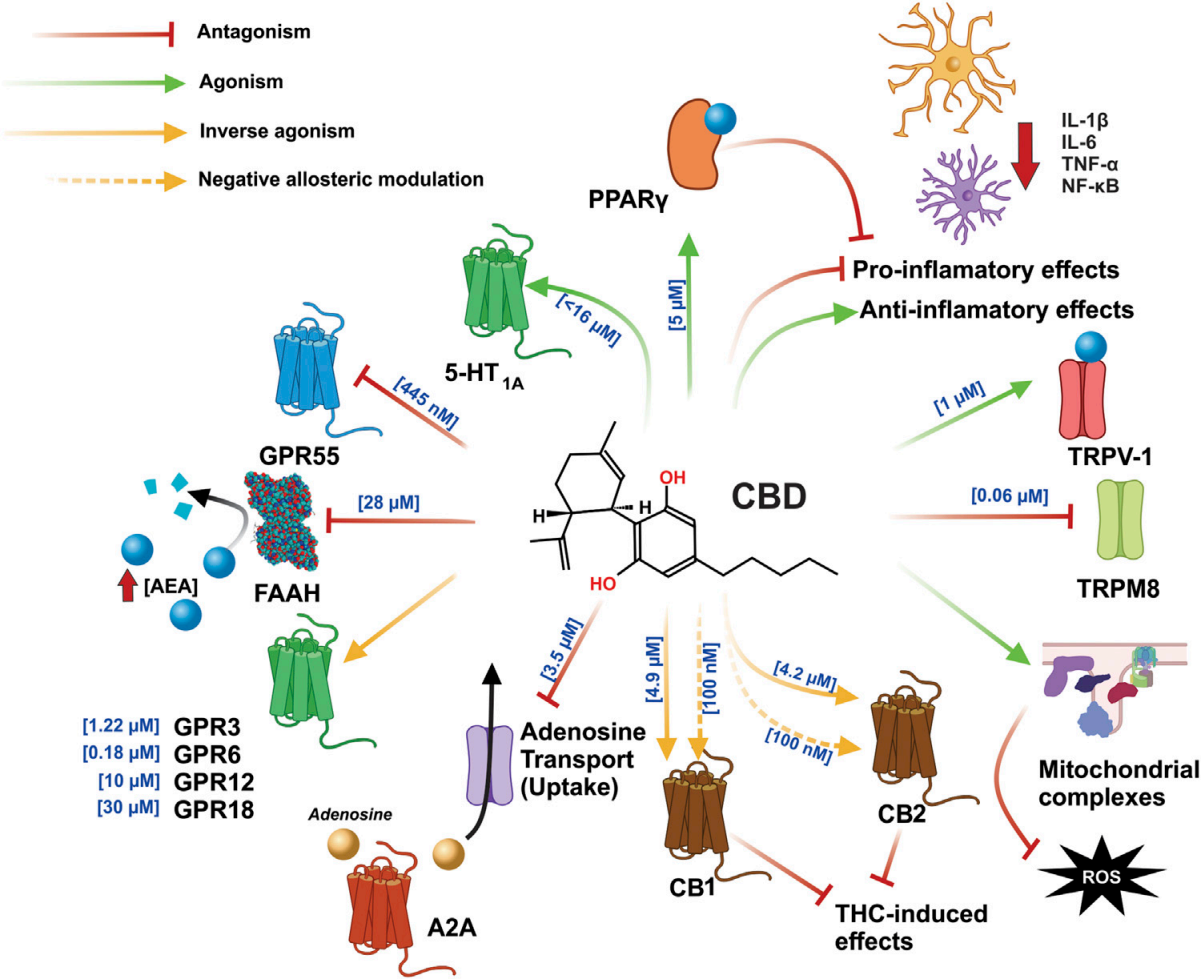
CBD exerts an agonist-like effect on the PPARγ, TRPV1, CB1, and CB2 receptors, by inhibiting the enzyme that degrades AEA and FAAH, leading to increased AEA concentration and greater interaction with said receptors. In addition, CBD inhibits GPR55 and TRPM8 and exerts an effect as an inverse antagonist on the GPR3, GPR6, GPR12, CB1, and CB2 receptors; moreover, in CB1 and CB2, it can function as a negative allosteric modulator, which is involved in blocking the effects of THC. The anti-inflammatory effects of CBD function by directly decreasing the synthesis of pro-inflammatory cytokines and increasing the synthesis of anti-inflammatory cytokines. CBD also reduces inflammation by stimulation PPARγ. Part of its antioxidant effects are achieved via the increased activity of mitochondrial complexes I, II, II-III, and IV. Abbreviations: CBD, cannabidiol; PPARγ, peroxisome proliferator activated receptor γ; TRPV1, transient potential receptor V1; CB1, cannabinoid receptor type 1; CB2, cannabinoid receptor type 2; AEA, anandamide; FAAH, fatty acid amide hydrolase; GPR55, G protein coupled receptor 55; TRPM8, transient potential receptor M8; GPR3, G protein coupled receptor 3; GPR6, G protein coupled receptor 6; GPR12, G protein coupled receptor 12; THC, tetrahydrocannabinol.

While the mechanism of action by which CBD exerts its therapeutic effects remains, to date, unclear, the interactions between CBD and various molecular targets can be divided into interactions that are either dependent on or independent of the ECS. The ECS-dependent effects of CBD occur via the CB1, CB2, and TRPV-1 receptors, as does its interaction with the FAAH enzyme. As CBD is a lipophilic structure, it is able to cross the blood-brain barrier (BBB) and thus modulate specific zones of the CNS (Calapai et al., 2020). While CBD was thought to have a low affinity for CBRs and able to activate them at high concentrations (>10 μM) (Howlett and Fleming, 1984), at low concentrations, it has been reported to act on the allosteric site of the CB1 receptor (Laprairie at al., 2015), which may maximize the binding of the orthosteric probe. At higher concentrations, meanwhile, this action may reduce the binding of the orthosteric probe, producing a bell-shaped curve (Tham et al., 2019). Cannabidiol acts as an inverse agonist on the CB1 and CB2 receptors, as demonstrated by the efficacy of its antagonist properties against the agonistic effects induced by CP55940 on the CB1 and CB2 receptors in [35S]GTPγS binding assays undertaken on membrane preparations (Thomas et al., 2007). The *K*_*B*_ values obtained for CBD as an antagonist of CP55940-induced [35S]GTPγS binding were 79 and 65 nM for CB1 and CB2, respectively, while the Ki values for the displacement of [3H]CP55940 were 4.9 and 4.2 µM for CB1 and CB2, respectively (Thomas et al., 2007). These findings have been supported by recent reports showing that CBD does not necessarily have to be present at the orthosteric site to act as an inverse agonist, meaning that it could induce a non-competitive negative allosteric modulation of the CB1 receptor (Laprairie et al., 2015). In addition, at 100 nM, CBD was found to be a negative allosteric modulator of CB2, with a Ki value of 4.2 µM observed for the displacement of WIN55212.2 (Martínez Pinilla et al., 2017). These findings demonstrate that cannabidiol is a high-potency antagonist of CBR agonists in the brain and has a negative allosteric modulatory effect (Figure 3).

It has also been reported that CBD is capable of inhibiting some of THC’s effects (Zuardi et al., 1982) by acting as a negative allosteric modulator on both CB1 and CB2 (Laprairie et al., 2015; Martí nez-Pinilla et al., 2017). Furthermore, computational models have identified an allosteric site on the CB1 receptor which is able to bind with CBD and, thereby, promote conformational changes to the receptor in either its active or inactive state. These findings may explain the negative allosteric modulatory effects of CBD on the CB1 receptor, with the possible participation of other molecular targets (namely independent interactions in the ECS) that, together, contribute to the effects observed in both *in vitro* and *in vivo* experimental studies (Chung et al., 2019). The mechanism by which CBD functions may be explained as a biased agonism, namely that it selects “which signaling pathways become activated upon binding to the receptor”. In 2018, it was reported that CBD, applied at 100 nM concentrations, produces a biased agonism effect against the effects of THC, by increasing cAMP levels and decreasing ERK ½ activity, thus countering the effects induced by the THC. These data may explain the controversial pharmacology of CBD, namely whether or not it interacts with cannabinoid receptors and results from the binding of the two receptors, CB1 or CB2, to allosteric sites (Navarro et al., 2018). In the ECS, CBD can exert an effect as an indirect agonist of the CB1 receptor by inhibiting both the FAAH enzyme and the AEA transporter (Bisogno et al., 2001; Ligresti et al., 2006; De Petrocellis et al., 2011), which leads to an increase in AEA levels and, consequently, in the activation of the CB1 receptor (Howlett et al., 2010), although it can also interact with CB2, TRPV1, and PPARɣ (Pertwee and Ross, 2002; Ross, 2003; Bouaboula et al., 2005). The TRPV-1 receptor is a molecular target for the pharmacological effects of CBD (Bisogno et al., 2001), which are highly potent (producing an EC50 of 1 µM) (De Petrocellis et al., 2011) and, via TRPV-1, increase Ca2+ levels (producing an EC50 of 0.7 µM) (Ligresti et al., 2006). Moreover, CBD, including its precursor, binds at [5 µM - >11.6 µM] and activates PPARγ at [10 –20 µM], while cannabidiolic acid (CBDA) binds at [7.6 µM] and activates PPARγ and is more effective than CBD in activating PPARγ at concentrations of [10 –25 µM] (O’Sullivan et al., 2009; Nadal et al., 2017) (Figure 3).

Notable among the independent mechanisms exerted by CBD in the ECS is the agonist binding of the G protein coupled receptors (GPCR) GPR3, GPR6, GPR12, and GPR18, which are considered orphan receptors (Morales and Reggio, 2017). However, lysophosphatidylinositol is considered an endogenous receptor for GPR55 (Alhouayek et al., 2018). CBD exhibits a decrease in concentration-dependent *ß*-arrestin two recruitment to both GPR3 and GPR6, but with greater potency for the latter (EC50 values of 1.22 and 0.18 μM, respectively) (Laun and Song, 2017). Furthermore, CBD significantly decreases the cAMP accumulation stimulated by GPR12, in a concentration-dependent manner, corresponding to an approximate EC50 of 10 μM (Brown et al., 2017). Therefore, these findings show the inverse agonist effect of the GPR3, GPR6, and GPR12 receptors. CBD is reported to present a low level of efficacy as an agonist in the recruitment, via GPR18, of *ß*-arrestin at a concentration of 30 µM (Console-Bram et al., 2014). However, CBD acts as an antagonist of the effects, induced by N-arachidonoyl glycine (NAGly) and THC, on the migration and morphology of microglia (McHugh et al., 2014). It is likely that the functionality of CBD as an agonist and antagonist depends on the expression of the GPR18 receptor and that CBD may also act as a biased agonist in this GPCR (Morales et al., 2020). It should be noted that, based on the inverse agonist effect that CBD has been shown to have on the GPR55 receptor, novel therapeutic strategies are proposed for treating neurodegenerative diseases via the probable mechanism of this phytocannabinoid (Ryberg et al., 2007; Kaplan et al., 2017). The GPR55 receptor is coupled to the Gα12/13 and Gαq proteins, while its activation promotes the release of Ca^2+^ stores from the endoplasmic reticulum and, in turn, the activation of MAPKs (Alhouayek et al., 2018). On the other hand, antagonism inhibits Ca^2+^ currents and causes neuronal inhibition at a presynaptic level. CBD is likely to promote both neuronal repolarization via GPR55, at a presynaptic level, and neuromodulation (Morano et al., 2020). Interestingly, as GPR55 is expressed in a similar way to the CB1 receptors, they are also expressed in the BG circuit (Celorrio et al., 2017).

In addition to the foregoing findings, evidence reported on the mechanism via which CBD affects the CNS has shown that CBD activates the serotonin receptor 5-HT1A (Russo et al., 2005) and the adenosine A2A receptors (Mecha et al., 2013). CBD behaves as an antagonist with TRPM8 (De Petrocellis et al., 2011), while, acting alone, it has also been found to stimulate the activity of mitochondrial complexes (Valvassori et al., 2013), in addition to, directly and indirectly, stopping the pro-inflammatory process and promoting the anti-inflammatory process, via PPARɣ (Esposito et al., 2011; Malfait et al., 2000) (Figure 3). It should be noted that the CB1 receptor, TRPV-1, GPR55, and the A2A receptor are all abundantly expressed in the BG, which are main structures that participate in the control of movement (Fernández-Ruiz et al., 2010a; Hickey and Stacy, 2012; Chaves-Kirsten et al., 2013; Celorrio et al., 2017).

## The Basal Ganglia in Parkinson’s Disease and the Neuromodulatory Role of Cannabidiol

**FIGURE 4:**
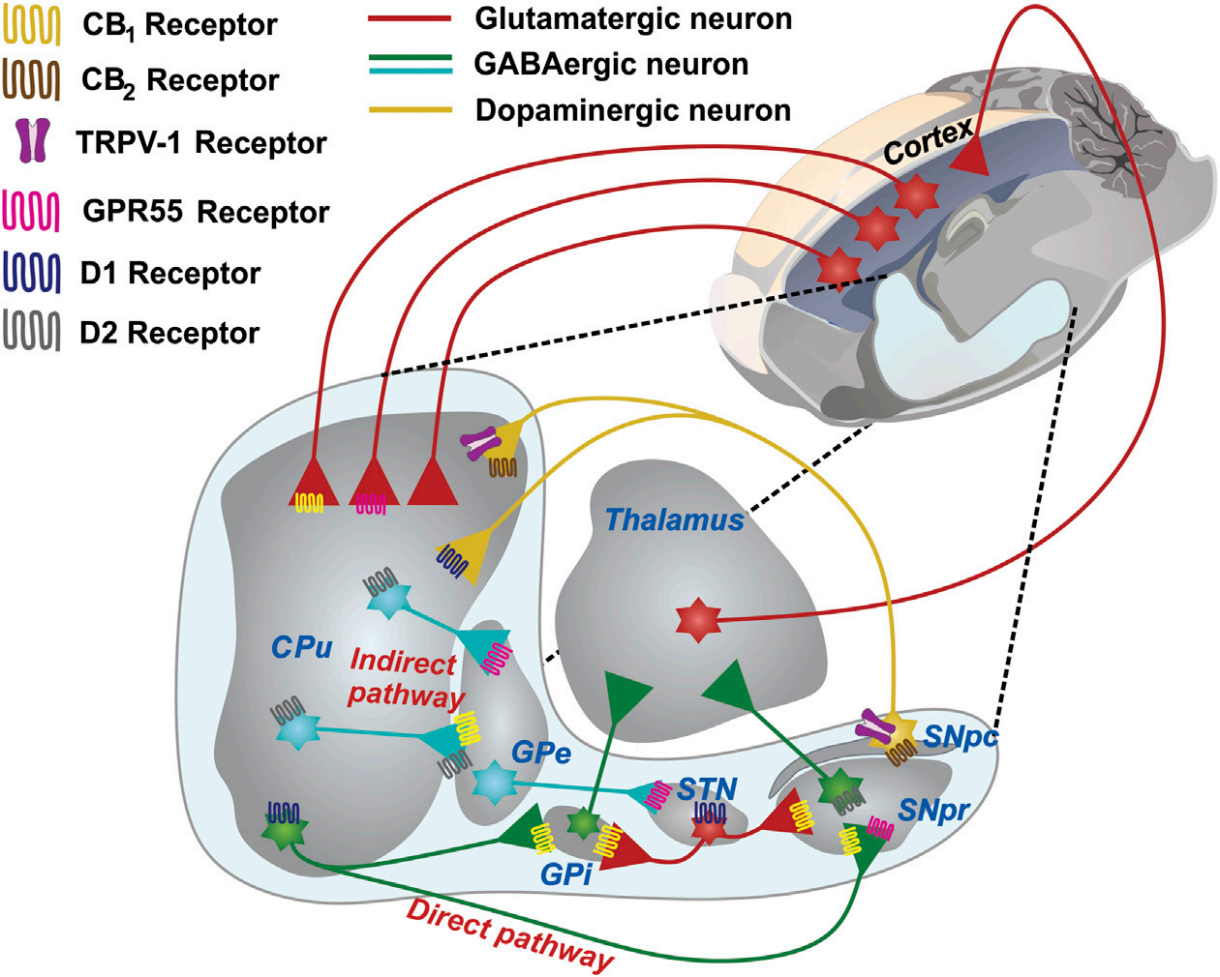
The basal ganglia network and its wide expression of receptors in presynaptic neurons. A schematic representing the GABAergic and glutamatergic connections in a sagittal section of the rat brain is shown. The CPu, the main input nucleus of the circuit, receives cortical projections of glutamatergic neurons. The expression of D1 receptors in the striatum forms the direct pathway of the BG circuit, which is projected toward the GPi and SNpr. The expression of the D2 receptors forms the indirect pathway, which is projected toward the GPe and subsequently toward the STN, which then sends projections to the GPi and SNpr. The projections that emerge from the exit nuclei direct the thalamus and return the processed information to the cerebral cortex. The expression of CB1 receptors mostly occurs in both GABAergic and glutamatergic neurons. GPR55 is present in the GPe, CPu, STN, and SNpc. CB2 and TRPV-1 are the sole in terminals of the SNpc. Abbreviations: CPu, Striatum or caudate-putamen; GPi, Internal *globus pallidus*; SNpr, *Substantia nigra pars reticulata*; SNpc, *Substantia nigra pars compacta*; GPe, External *globus pallidus*; STN, subthalamic nucleus.

The BG are subcortical nuclei that constitute a parallel and partially closed circuit and are brain structures essential for promoting the initiation and execution of voluntary movements (Lanciego et al., 2012; Klaus et al., 2019). The following nuclei make up the motor circuit of the BG: the caudate-putamen (CPu), which is known as the striatum in rodents and is the main entry nucleus of the BG; the internal and external *globus pallidus* (GPi and GPe, respectively); the subthalamic nucleus (STN); and, the *substantia nigra pars reticulata* and *substantia nigra pars compacta* (SNpr and SNpc, respectively). The main exit nuclei of the BG are the GPi and SNpr (Albin et al., 1989; Lanciego et al., 2012). The neural information that enters the circuit mainly arrives from the sensorimotor cortex and then circulates through the BG and the thalamus, finally returning to the cerebral cortex. (Alexander and Crutcher, 1990; Haber and Calzavara, 2009). This loop plays an important role in guiding motor behavior; however, the release of dopamine from SNpc neurons in the entry nucleus of the BG is necessary for the motor circuit to properly function (Gerfen and Surmeier, 2011). Various movement disorders emerge from neurochemical dysfunction in the CPu, with dopamine deficiency and the loss of dopaminergic neurons comprising the main characteristics of PD (Figure 4).

Parkinson’s disease is a chronic and progressive neurodegenerative condition that manifests in people with characteristic clinical symptoms, such as tremor at rest, bradykinesia, muscle stiffness, and postural instability (Clarke, 2007). Parkinson’s disease is the second most common neurodegenerative disease in the world, manifesting in people over 60 years of age (Mhyre et al., 2012), with a global prevalence of 5–35 cases per 100,000 people (Savica et al., 2017). Since the discovery of the decrease in dopamine in the striatum of patients with PD in the 1960s (Hornykiewicz, 1962), the treatment of choice for the disease has been the administration of l-DOPA (Carlsson et al., 1957; Birkmayer and Hornykiewicz, 1961; Bogetofte et al., 2020). However, the chronic administration of l-DOPA in parkinsonian patients shows choreatetotic-type motor complications, called l-DOPA-induced dyskinesia (LID). (Tran et al., 2018). As it has been proposed that this movement disorder, is more disabling than the disease itself, pharmacological proposals for reducing LIDs, such as the use of CBD, could improve quality of life for the parkinsonian patient.

With neurochemical and therapeutic findings showing that dopamine is a key regulatory neurotransmitter in the motor circuit of the BG, the activation of the dopaminergic system is generally associated with increased movement, while its inhibition is associated with hypokinesis (Fernández-Ruiz et al., 2010a). Dopaminergic deficit in the striatum is associated with morphological changes across all BG, a decrease in the number of dendritic spines of the medium spiny neurons (MSNs) in the striatum, and alterations in the neuronal connectivity of the striatopallidal pathway (an indirect pathway) and the *striatum-nigral* pathway (a direct pathway) (Galvan, et al., 2015).

Due to the fact that the CB1 receptor is found at the presynaptic level, its activation promotes neuromodulatory action via retrograde eCB signaling, mainly in the synapses located in those brain structures that regulate the motor process, namely the corticostriatal pathway and the direct and indirect pathways of the BG (Covey et al., 2017). This action is significant for the functioning of the excitatory and glutamatergic neurons which carry neuronal information through the cortex to the CPu, while the neurons that carry information from the CPu to the output nuclei are inhibitory and GABAergic in nature (DeLong, 1990). The neurotransmitter dopamine is closely related to the action performed by cannabinoids (Fernández-Ruiz et al., 2010a; Covey et al., 2017). The CB1 receptor has been considered to be the main receptor involved in controlling the synaptic activity of the dopaminergic neurons of the *nigro-striatal* pathway, although these neurons do not express CB1 as well as other subpopulations of dopaminergic neurons, such as the mesostriatal pathway and the cortico-limbic system (García et al., 2016). However, the expression of the CB2 receptor has recently been reported in the dopaminergic neurons of the ventral tegmental area, an important neuronal area that modulates reward (Liu et al., 2017). In human brains, the expression of CB2 in the *nigro-striatal* pathway (García et al., 2015) has been demonstrated and has even been shown to be associated with pathological conditions (Cassano et al., 2017). The specific deletion of CB2 from the dopaminergic neurons of DAT-Cnr2 conditional knockout (cKO) mice has shown that CB2 may play an important role in modulating psychomotor behaviors, anxiety, and depression, as well as the rewarding effects of alcohol and cocaine. Furthermore, human genome-wide association studies have shown that the Cnr2 gene is associated with PD and substance abuse disorders (Liu et al., 2017). Therefore, the regulation that the CB2 receptor may exert on dopaminergic neurons and that which the CB1 receptor may exert on the GABAergic neurons of the striatum, GPi, and SNpr could be crucial for neuroprotective and neuromodulatory cannabinoid therapy using CBD (Figure 4).

The effects observed when the CB1 receptor is activated or blocked in the BG circuit are caused by its action on other neuronal populations, such as the GABAergic (da Silva et al., 2015), glutamatergic (Ren et al., 2009), or opiodergic populations (Prud’homme et al., 2015), which comprise neurons interconnected with the dopaminergic neurons (Stampanoni Bassi et al., 2017). However, it should be noted that anandamide (AEA), N-arachidonoyl-dopamine (NADA), and the synthetic compound AM404 interact and activate the TRPV1 receptor, which is expressed in the *nigro-striatal* pathway, thus enabling the direct activation of eCB in the dopaminergic system (Mezey et al., 2000; Cristino et al., 2006) (Figure 4). The CB1 receptors are capable of forming heterodimers with the D2 receptors in striatal projection neurons, enabling both systems to interact directly in postsynapse (Blume et al., 2013). Therefore, the range of receptors found in the striatum may be involved in the modulation, via eCB and cannabinoids such as CBD, that is proposed in the present study.

A study conducted on intact rat striatal synaptosomes identified various modulatory mechanisms that cannabinoids may execute on the reuptake of dopamine, glutamate, and adenosine (Pandolfo et al., 2011). Specifically, CBD has shown a low capacity for inhibiting dopamine reuptake (IC50 = 16.5 µM), a finding similar to that reported by Poddar and Dewey 1980, who found that, in striatum synaptosomes, high concentrations of CBD were needed to produce an inhibitory effect on dopamine recapture. This finding is important for understanding the modulatory role of CBD in PD, as 90% of dopaminergic neuronal death occurs in PD (Cheng et al., 2010), meaning that CBD treatment in the latter stages of the disease is likely to be ineffective. Furthermore, CBD had potent inhibitory effects on adenosine reuptake (IC50 = 3.5 µM) (Pandolfo et al., 2011), which may explain its neuromodulatory activity via the expression of the A2A receptors in the BG circuit (Schiffmann et al., 2007). The A2A receptor has been shown to be widely expressed in the striatopallidal pathway, in presynaptic and postsynaptic GABAergic neurons (Rosin et al., 2003; Diao et al., 2017). Moreover, as the function of the A2A receptor is to inhibit the release of GABA, it will promote movement in an animal PD model (Schwarzschild et al., 2006). However, it is likely that interactions with various neurotransmitter receptors can activate, in addition to neuronal modulation, neuronal signaling pathways that promote neuronal survival. To address the probable neuroprotective effect of CBD, it is necessary to identify the proposed pharmacological approaches that harness the medicinal properties of phytocannabinoids as an adjuvant in PD treatment.

## Cannabidiol as an Adjuvant in Parkinson’s Disease Treatment

Various studies have suggested that both genetic (5–10%) and idiopathic factors may contribute to the neurodegeneration that occurs in *p*D. However, the etiology of this disease, namely the underlying cause of dopaminergic neuronal death, is unknown (Kalinderi et al., 2016; Deng et al., 2018). Several studies have linked the idiopathic factors in PD to both aging and environmental factors (heavy metals, pesticides, head trauma, and viral infections) (Ascherio and Schwarzschild, 2016; Pang et al., 2019). Two processes, oxidative stress and neuroinflammation, are closely related to both the genetic and idiopathic factors observed in PD (Hald and Lotharius, 2005). There is evidence that the dopaminergic neurons of the SNpc are vulnerable to oxidative damage, as they present low levels of antioxidant enzymes, such as glutathione peroxidase, and high levels of pro-oxidants, such as free iron and neuromelanin (González-Hernández et al., 2010). The oxidative characteristics of the SNpc promote increased ROS levels, induce the inhibition of the mitochondrial electron transport chain, increase glutamate levels, stimulate NMDA receptors, and, finally, induce the processes of excitotoxicity and neuronal death (Hernandez-Baltazar et al., 2019). Indeed, one of the aims of neuroprotective therapeutic strategies for PD is to reduce the cytotoxic effects of oxidative stress, namely lipid peroxidation, protein nitration, and DNA oxidation, a point reviewed in the next section.

**FIGURE 5:**
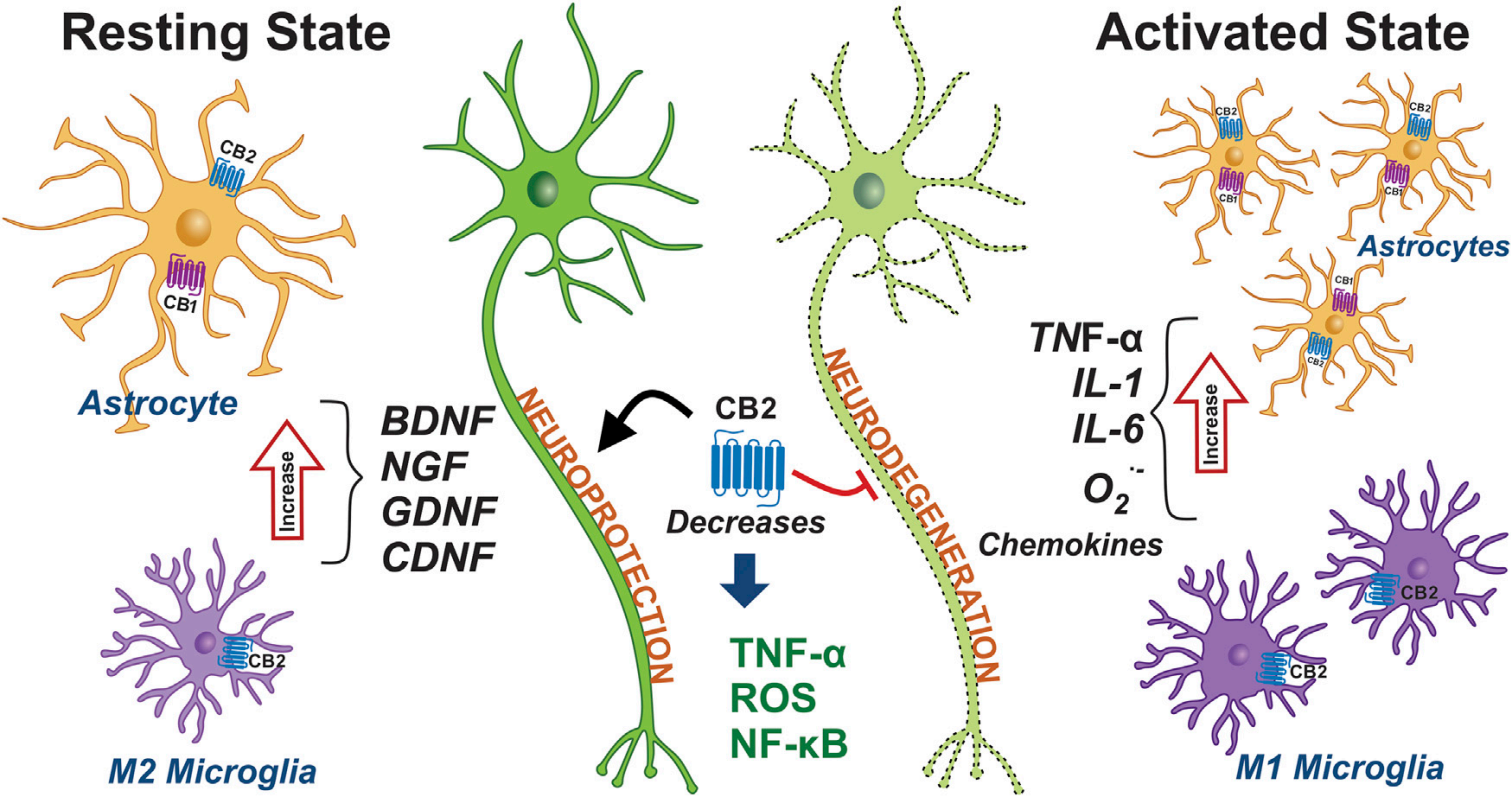
Expression of cannabinoid receptors in glial cells and their role in PD. The phenotypes of microglia and astrocytes are schematically represented under non-inflammatory conditions, namely in a resting state. The microglia and astrocytes are shown in pro-inflammatory conditions and in an active state. The activation of microglia and astrocytes promotes and triggers the neuroinflammation that contributes to *P*D. The activation of the CB1 and CB2 receptors may decrease the inflammation seen in *P*D. Abbreviations: CB1, cannabinoid receptor type 1; CB2, cannabinoid receptor type 2; BDNF, brain-derived neurotrophic factor; NGF, nerve growth factor; GDNF, glial cell line-derived neurotrophic factor; CDNF, cerebral dopamine neurotrophic factor; ROS, reactive oxygen species; NF-κB, nuclear factor kappa B; IL-1, interleukin-1; IL-6, interleukin-1; O_2_∙^-^, Superoxide anion; TNF-α, tumor necrosis factor alpha.

In addition to the role played by the neuronal population, the participation of glial cells (astrocytes and microglia) is essential for the development of PD (Hernandez-Baltazar et al., 2019; Domingues et al., 2020). Glial cells are associated with neuroinflammation and the neurodegenerative process, with the former characterized by reactive microglia and the presence of astrocytes alongside neurons with dopaminergic injury (Bachiller et al., 2018). Microglia are considered the resident innate immune cells and are, therefore, capable of robust chemotaxis, phagocytosis, and cytokine production and release (Domingues et al., 2020). It should be noted that recent studies have phenotypically categorized microglia cells into M1 (pro-inflammatory) and M2 (anti-inflammatory) states (Tang and Le, 2016) (Figure 5). In the M2 state, microglia improve neuronal survival by releasing glial cell line-derived neurotrophic factor (GDNF) (Ding et al., 2004) and are involved in the upregulation of tissue repair and gene regeneration (Le et al., 2016). In contrast, in the M1 state, microglia promote the neurodegeneration of the nigrostriatal pathway in *p*D. Microglia produce and increase ROS levels in a pro-inflammatory state, thus producing IL-1β, IL-6, TNFα, chemokines, NO^**?**^ **,** and O_2_^**∙**-^ (Liu and Hong, 2003; Subramaniam and Federoff, 2017). This release of pro-inflammatory cytokines activates signaling pathways in order to promote microglia recruitment and, thus, dopaminergic cell death (Hernandez-Baltazar et al., 2019).

Astrocytes, the class of glial cells that are most present in the mammalian CNS, are metabolic, supportive of neuronal structure, and release neurotrophic factors. Furthermore, they maintain the integrity of the BBB and modulate neuronal transmission and excitability (Domingues et al., 2020). Following dopaminergic neuronal injury, mature astrocytes proliferate and promote neuronal regeneration via brain-derived neurotrophic factor (BDNF) and cerebral dopamine neurotrophic factor (CDNF) (Hernandez-Baltazar et al., 2019). Astrocytes detect cellular insult signals and trigger an immune response through the secretion of cytokines and chemokines. However, an imbalance in the secretion of pro-inflammatory/anti-inflammatory substances contributes to neuroinflammation and chronic neurodegeneration (Colombo and Farina, 2016; Liddelow et al., 2017). For this reason, one of the novel and opportune treatment strategies for PD is to modulate the neuroinflammation occurring during the progression of the disease.

Interestingly, while both the CB1 and CB2 receptors are expressed in astrocytes, the CB2 receptor is overexpressed under neuroinflammatory conditions in both the microglia and astrocytes (Benito et al., 2005; Cassano et al., 2017). Although the main receptor involved in the modulation of reactive glia is the CB2 receptor, a neuroprotective effect of the CB1 receptor that directly involves the glia cannot be ruled out (Chung et al., 2011). The modulating effects of astrocytic activity with brain injury is mediated by cannabinoids via the CB2 receptor, or the CB1 and CB2 receptors combined (Fernández-Ruiz et al., 2010a; Stella, 2010) (Figure 5). These effects promote a trophic role or provide anti-inflammatory mediators that can rescue damaged neurons (IL-10, TGF-β) and promote the reduction of chemokine levels by astrocytes such as fractalkine, an effect that would be predominantly mediated by the activation of CB2 receptors (Smith et al., 2000; Molina-Holgado et al., 2003). Particularly when activated, microglia are affected by the activation of CB2 receptors in the CNS, while the CB2 receptors also play a role in the proliferation and migration of these cells at injury sites (Walter et al., 2003; Carrier et al., 2004). The activation of CB2 receptors in microglia dampens the generation of neurotoxic factors such as TNF-α (Stella, 2010) and the transcription factor NF-κB, which regulates pro-inflammatory responses (Oh et al., 2010). Therefore, the expression of the CB2 receptor in both reactive microglia and astrocytes suggests that it could be a target for promoting neuroprotection (Fernández-Ruiz et al., 2015). It is likely that CBD is able to regulate the CB1 and CB2 receptors in both the glial cells and the BG circuit, via non-canonical mechanisms.

Currently, orthodox l-DOPA therapy reduces the symptoms of PD; however, there are no therapies that can prevent or rescue neurons from death (Choonara et al., 2009; Schapira et al., 2014). In these circumstances, l-DOPA is no longer metabolized by dopaminergic neurons, due to the degeneration of between 50 and 70% of nigral DA neurons (Carta and Bezard, 2011). Serotonergic neurons possess the enzymatic machinery for synthesizing dopamine via l-DOPA, promoting vesicular storage, and expressing the vesicular monoamine transporter (VMAT). However, serotonergic neurons lack a regulatory mechanism for dopamine release and regulation, a function which is carried out by the presynaptic D2 receptor and, therefore, induces the excessive release of dopamine into the CPu (Arai et al., 1995). As the disease progresses and dopaminergic neuronal death increases, the efficiency of l-DOPA decreases and patients experience the abnormal involuntary movements known as LIDs (Putterman et al., 2007; Francardo et al., 2011). For this reason, it is necessary to develop new non-dopaminergic drugs capable of reducing or attenuating motor symptoms without inducing dyskinesias, with the use of cannabinoids an interesting therapeutic approach to PD, one which has emerged alongside a new class of drugs. Cannabidiol has no psychoactive effects and has shown encouraging results in preclinical and clinical trials conducted on different neurodegenerative diseases. It is also a multi-target drug, as, in addition to acting on the ECS, it can act on the serotonin, adenosine, dopamine, and opioid receptors (Russo et al., 2005; Carrier et al., 2006; Kathmann et al., 2006; Thomas et al., 2007; Pandolfo et al., 2011; Linge et al., 2016; Sonego et al., 2018). As most of the aforementioned CBD-activated receptors are coupled to an inhibitory G protein, they are capable of acting as neuromodulators, given that they regulate the release of other neurotransmitters.

Clinical trials evaluating the effects of cannabinoids on PD show conflicting results. Nabilone (a non-selective CB1 receptor and CB2 receptor agonist) decreases l-DOPA-induced dyskinesias. It has been suggested that the lateral *globus pallidus* (GPl) exbibits hyperactive behavior in the dyskinetic process and that the stimulation of CBRs improves GABAergic transmission by reducing GABA reuptake in the GPl (Sieradzan et al., 2001). The oral administration of a cannabis extract in PD patients was well tolerated but did not produce an anti-parkinsonian effect (Carroll et al., 2004), while the administration of cannabis has been shown to have a beneficial effect on tremor and stiffness, a minor effect on bradykinesia, and a tendency to improve posture, all of which are motor symptoms of *p*D. Cannabis been found to have a positive impact on non-motor symptoms, such as sleep and pain (Lotan et al., 2014), with the latter finding potentially attributable, in part, to nighttime pain relief and, in part, to the drug’s calming and soporific effects. Some studies have found cannabis-induced improvements in sleep quality rather than motor symptoms (Finseth et al., 2015). While a single smoked dose of marijuana has not been found to decrease tremor in PD patients, its sedative or anxiolytic effect benefits some patients when anxiety is a significant trigger (Frankel et al., 1990). The different results obtained by these studies are related to variations in the amount of plant extract administered and the different routes of administration, where, for example, oral administration produces lower plasma concentrations than inhalation.

A double-blind, placebo-controlled, cross-sectional study evaluated the severity and duration of LIDs and the effect on these symptoms of the administration of a cannabis extract (Cannador^®^), comprising THC/CBD, to eighteen patients, in doses of up to 0.5 mg/kg/day. The results obtained did not show any significant difference, although some patients did report reduced tremor and improved sleep quality compared to those who had received the placebo, as well as an improved dementia score via the Mini-Mental State Examination (MMSE) (Carroll et al., 2004). Some of the first clinical studies in this area were driven by testimonies, such as the anecdotal account of a patient with severe Parkinson’s tremor who had been resistant to different types of medication until experiencing dramatic relief from smoking marijuana. This prompted a study to be conducted on five idiopathic PD patients, who were evaluated via the Webster scale and revealed improvements in tremor after the administration of smoked marijuana (1 g ≈ 29 mg of THC), l-DOPA, and apomorphine. These authors found no evidence to show that smoking cannabis reduces tremor or other parkinsonian symptoms (Frankel et al., 1990). Another evaluation of smoked cannabis was undertaken in an open study, in which, of the 22 patients who received ultimately ineffective treatments to relieve pain and tremor for ten months, seven experienced motor fluctuations. Moreover, an improvement in motor symptoms was obtained, with a greater benefit observed in tremor and bradykinesia, while, in addition to improved posture, improvements in non-motor symptoms and good tolerability were also found (Lotan et al., 2014).

Recent studies have highlighted the synergistic effect among the components of cannabis (Samarut et al., 2019), especially those with the highest concentrations, namely THC and CBD (Jin et al., 2020). This effect may generate limitations, as it is difficult to determine the mechanism by which results were obtained, although cannabis also has advantages over other treatments due to its benefits in terms of various therapeutic objectives, mainly achieved via the use of CBD (Morales et al., 2017; Peres et al., 2018). As the improvement of parkinsonian symptoms was observed to be related to the use of cannabis for more than two months (Venderová et al., 2004), the time period in which the treatment is applied is an important factor. More studies are required in order to evaluate the effect of cannabinoids on LIDs, considering the duration, the dosing, and the use of routes of administration that do not cause secondary damage (Varlet et al., 2016; Millar et al., 2018).

## Cannabidiol As A Drug With Probable Neuroprotective Properties

The first reports of clinical research on the treatment of PD with CBD were followed by the studies carried out by Snider and Consroe (1985) and Consroe et al. (1986). They showed that, in two patients with dystonia and coexisting Parkinsonian characteristics, oral CBD treatment at doses higher than 300 mg/day exacerbated hypokinesia and tremor at rest, and had a positive effect on dystonic movements (Snider and Consroe, 1985). Consroe showed CBD to be effective in treating LIDs in PD patients, a finding that was perhaps the first to show the beneficial effect of CBD on LIDs (Consroe et al., 1986); however, given that he did not report the beneficial effects of CBD on PD, it is likely that interest in the potential of CBD as a treatment for this condition dwindled. It was not until the 2000s that CBD regained relevance in PD research, as a result of a study conducted on healthy recreational users of both marijuana (plant) and resin (hashish). The study analyzed subjects’ hair samples for both THC and CBD levels (as determined by chromatography/mass spectrometry), finding an increase in the levels of the markers of the cerebral metabolism of N- Acetylaspartate (NAA)/Total Creatinine (tCr) in the putamen/*globus pallidus*, as determined by magnetic resonance spectroscopic imaging (MRSI). A positive correlation between NAA/tCr and CBD was also observed in the striatopallidal pathway (Hermann et al., 2007), a finding which may reflect a possible improvement in the neuronal and axonal integrity of the indirect BG pathway due to the effects of CBD. In addition, this finding led to the proposal of CBD as a therapeutic target during the initiation of PD, given that GPe is the nucleus with the highest expression of the CB1 receptor and is the nucleus with the highest level of GABAergic activity, thus promoting hypokinesia.

Studies on the administration of CBD have been undertaken on patients with non-motor PD symptoms (Table 1). In 2009, Zuardi carried out a study on six patients presenting both psychosis and the motor symptoms of *p*D. The four-week treatment regime began with a 150 mg dose, which, depending on the clinical response, was increased by 150 mg each week. All evaluations were performed via tests and clinical evaluation scales for anxiety and cognition, with CBD observed to decrease PD psychosis, while no difference was observed in motor processes (Zuardi et al., 2009). The same author conducted two parallel studies evaluating PD motor disorders and REM sleep behavior disorder (RBD). The effect of the CBD treatment on RBD was evaluated in a group of four patients with symptoms characteristic of PD and the sleep disorders caused by the disease. The CBD dose was 75 mg/day and 300 mg/day per patient, with both treatments lasting six weeks. The polysomnograph evaluation conducted revealed that the CBD attenuated RBD (Chagas et al., 2014a).

**TABLE 1:** Clinical research reports on the effect of CBD on Patients of PD.

Patient characteristics	Symptoms	CBD dosage and temporary treatment	Medical evaluations and study techniques	Main findings	Author (reference)
13 male recreational cannabis users (six users consumed marijuana, three hashish and four marijuana and hashish). Mean age 22 years, approximately	All participants were medication free. They had not brain disorders and other diseases	6 years of using marijuana or hashish	Chromatography/mass spectrometry (GC/MS) to hair analysis of cannabinoids (THC and CBD) ^1^H magnetic resonance spectroscopic imaging (MRSI) markers of brain metabolism: NAA, cho and tCr	↑ positive correlation of NNA/tCr and CBD in the putamen/*globus pallidus*	Hermann et al. (2007)
Six patients (four men and two women). Mean age 58 years, approximately	Patients had psychosis for 3 months and motor sympotms of PD	150 mg CBD (p.o.), and increasing 150 mg every week depending on the clinical response. Four weeks of treatment	Bech’s version of the brief psychiatric rating scale (BPRS) structured interview guide with test–retest reliability of the BPRS Parkinson psychosis questionnaire (PPQ) unified Parkinson’s disease rating scale (UPDRS) clinical global impression – Improvement scale (CGI–I) Mini-Mental State Examination (MMSE) frontal assessment battery (FAB)	↓ psychotic symptoms in PD CBD did not worsen the motor function CBD did not induce any decrease in cognitive function	Zuardi et al. (2009)
Four male PD patients with RBD. Mean age 63 years, approximately	Alterations during sleep characterized by swearing, talking, yelling, pushing, kicking, punching and gesturing and motor sympotms of PD	75 mg/day CBD (p.o.) in three patients 300 mg/day CBD (p.o.) in one patient duration of treatment, six weeks	Polysomnography (PSG) periodic limb movement index (PLMI)	↓ frequency of RBD-related events	Chagas et al. (2014a)
21 (15 male and 6 female) PD patients. Mean age 65 years, aproximately	Motor symptoms of idiopathic PD, score between 1 and 3 in the hoehn and yahr scale	75 mg/day or 300 mg/day CBD (p.o.) duration of treatment, six weeks	UPDRS to assess PD symptoms Parkinson'|’s disease questionnaire – 39 (PDQ-39) plasma levels of BDNF (ELISA) proton magnetic resonance scans (MRS)	↑ functioning and well-being of PD patients (NC) motor score evaluated with the UPDRS (NC) BDNF plasma levels (NC) NAA/Cr for MRS	Chagas et al. (2014b)
24 (male and female) idiophatic PD patients	Motor symptoms of idiopathic PD, and anxiety an absence of marked cognitive alterations	300 mg CBD (p.o.) with interval between the first and the second experiment was 15 days (two administration)	UPDRS to assess PD symptoms hoehn and yahr scale schwab and england scale simulated public speaking test (SPST)- VAMS; SPST; SSPS systemic blood pressure and heart rate tapping test accelerometer (tremors measured)	↓ SPST-induced anxiety ↓ tremor amplitude in patients with PD	De Faria et al., 2020

BDNF, brain-derived neurotrophic factor; CBD, cannabidiol; Cho, choline; ELISA, enzyme-linked immunosorbent assay; NAA, N-acetylaspartate; RBD, REM sleep behavior disorder; SPST, simulated public speaking test; SSPS, self-statements during public speaking scale; tCr, total Creatine; THC, tetrahydrocannabinol; VAMS, visual analog mood scales; (↑) increase; (↓) decrease; NC, no changes.

In order to explore the role of CBD in the motor symptoms of PD, a study was conducted with 21 PD patients who had recorded a score of one to three on the Hoehn and Yahr scale, using a CBD dose of 75 mg/day or 300 mg/day for three weeks. While an increase in general well-being and functioning in daily tasks was observed, alterations were not observed in the motor score evaluated via the Unified Parkinson’s disease Rating Scale (UPDRS) or the plasma levels of BDNF and NAA/Cr, as measured via proton magnetic resonance scans (MRI) (Chagas et al., 2014b). The results for effects of CBD on PD in clinical research are likely not as encouraging, suggesting that increased clinical research efforts are required in this area.

Hampson et al. first reported the neuroprotective effects of CBD in 1998, using the primary cultures of cortical neurons exposed to toxic concentrations of the neurotransmitter glutamate [250 µM] for 10 min, finding that CBD prevented both glutamatergic neurotoxicity, with an EC50 of 2–4 μM, and cell death induced by oxidative stress. In addition, they observed that the antioxidant effect of CBD was more powerful than *a*-tocopherol and ascorbate in equimolar concentrations, finding that neuroprotection was not inhibited by CBR antagonism, thus indicating the independent therapeutic effects of CB1 and CB2 (Hampson et al., 1998). The CBR-independent antioxidant effect of CBD has also been demonstrated in B-lymphobalstoid and fibroblast cell cultures serum-starved for survival (Chen and Buck, 2000). The ability of CBD to attenuate the neurotoxicity induced by two oxidative insults was also observed, finding stress and mitochondrial dysfunction in cultured granular neurons at 2.5 μM concentrations due to the effect of both CBR-independent and 5-HT-1A mechanisms (Echeverry et al., 2020). As the antioxidant effect of CBD was observed to play an important role in neuroprotection, it was proposed as a possible therapeutic agent in the treatment of highly oxidative neurodegenerative disorders, such as PD.

The animal PD models most frequently used in preclinical research are administered neurotoxic agents such as 6-OHDA (mostly used in rats and mice) and MPTP (mostly used in monkeys and mice) (Duty and Jenner, 2011; Blesa et al., 2012). One of the goals of using an animal PD model is to study the drugs that prevent dopaminergic neuronal death. Attempts have been made to delay, or even arrest, dopaminergic degeneration with different chemicals, such as synthetic antioxidants (Moosmann and Behl, 2002), N-Methyl-d-aspartate receptor antagonists (NMDA) (Alexi et al., 2000; Olivares et al., 2012), Ca^2+^ channel blockers (Rodnitzky, 1999; Kang et al., 2012), and anti-inflammatory substances (McGeer et al., 2001; Martinez and Peplow, 2018). However, these pharmacological strategies, which aim to treat the main predisposing factors for PD, are yet to yield satisfactory neuroprotection results.

The proposal of phytocannabinoids as a potential promoter of dopaminergic neuroprotection in animal PD models began with the work of Lastres-Becker et al. (2005) (Table 2), who administered THC and CBD every 24 h for 2 weeks at a dose of 3 mg/kg i.p., one day after injury with 6-OHDA in the medial bundle of the forebrain. They showed that THC and CBD play a neuroprotective role by decreasing dopaminergic neuronal death, although it should be note that CBD increased dopamine concentrations in the striatum. While a modification of the expression of the CB1 and CB2 receptors was not found in the 6-OHDA model, decreased TRPV1 receptor expression, a receptor expressed in the *nigro-striatal* pathway, was observed (Mezey et al., 2000).

**TABLE 2:** Preclinical research reports of effect CBD on various *in vivo* models of PD.

Animal species studied	Lesion model	Lesion time	CBD dosage	Treatment time	Brain nuclei studied	Biochemical markers	Proteins/mRNA evaluated	Evaluated animal behavior	Author (reference)
Sprague–Dawley rat	6-OHDA [8 μg/2 μl] in MFB	2 weeks	CBD (3 mg/kg i.p.)	Two weeks, CBD administration 16 h post 6-OHDA	Striatum	↑ dopamine (NC) DOPAC. (NC) TH activity		Not evaluated	Lastres-Becker et al. (2005)
					SNpc		(NC) mRNA SP (ISH) (NC) mRNA PENK (ISH) (NC) mRNA TH (ISH)		
Sprague–Dawley rat	6-OHDA [8 μg/2 μl] in MFB	2 weeks	CBD (3 mg/kg i.p.)	1 week (1W), CBD administration 7 days' post 6-OHDA 2 weeks (2W), CBD administration 16 h post 6-OHDA	Striatum	↑ dopamine 2W (NC) dopamine 1W		Not evaluated	García-Arencibia et al. (2007)
					SNpc		↑ mRNA SOD Cu/Zn 2W (ISH) (NC) mRNA SOD Cu/Zn 1W (ISH)		
Sprague–Dawley rat	6-OHDA [200 μg/5 μl] i.c.v injection	2 weeks	CBD enriched botanical extract (equivalent to 3 mg·kg-1 of pure CBD i.p.)	2 weeks, CBD administration 16 h post 6-OHDA	SNpc	Not evaluated	↑ TH (IHC) ↓ OX-42 (IHC)	Not evaluated	García et al. (2011)
Wistar rat	Reserpine (dosage 1 mg/kg s.c.) 2 days administration	6 days	CBD (0.5 and 5 mg/kg i.p.)	7 days, administration one day before reserpine	Not evaluated	Not evaluated	Not evaluated	(NC) locomotor activity ↓ time bar test catalepsy ↓ vacuous chewing movements plus-maze discriminative avoidance task (attenuate the reserpine-induced memory deficit)	Peres et al. (2016)
C57BL/6Mice	MPTP (dosage 20 mg/kg i.p.)	5 weeks	CBD (5 mg/kg i.p.)	5 weeks. Joint administration with MPTP	Striatum	Not evaluated	(NC) TH (IHC) (NC) Iba-1 (IHC)	(NC) time to descended in pole test ↓ latency to fell in rotarod (CBD in animals control)	Celorrio et al. (2017)
					SNpc		(NC) TH (IHC)		
	Haloperidol (dosage 1 mg/kg i.p.)	180 min induced catalepsy	CBD (5 mg/kg and 20 mg/kg i.p.)	60 min, administration 2 h after injection of haloperidol	Not evaluated	Not evaluated	Not evaluated	(NC) time in the bar test catalepsy	
C57BL/6Mice	6-OHDA [5 μg/2 μl] dorsolateral striatum injection	21 and 38 days	CBD (10 mg/kg i.p.)	CBD chronic administration, 14 days; 24 days post 6-OHDA	Not evaluated	Not evaluated	Not evaluated	↑ latency in tail flick test	Crivelaro do Nascimento et al. (2020)
			CBD (10 mg/kg i.p.)	Acute administration, a single injection at 21 days' post 6-OHDA.				↑ latency in tail flick test ↑ latency hot plate test ↑ latency to mechanical stimulus in von frey test ↑ latency to cold stimulus in acetone drop test	
			CBD (30 mg/kg i.p.)					↓ latency to mechanical stimulus in von frey test ↓ latency to cold stimulus in acetone drop test	
			CBD (100 mg/kg i.p.)					↑ latency to mechanical stimulus in von frey test ↑ latency to cold stimulus in acetone drop test	

6-OHDA, 6-hydroxydopamine; CBD, cannabidiol; DOPAC, dihydroxyphenylacetic; i.c.v, Intracerebroventricular; Iba-1, ionized calcium-binding adaptor protein-1; IHC, immunohistochemistry; ISH, *in situ* hybridization; MFB, medial forebrain bundle; MPTP, 1-methyl-4-phenyl-1,2,3,6-tetrahydropyridine; OX-42, oxycocin-42; PENK, proenkephalin; SNpc, substantia nigra pars compacta; SOD Cu/Zn, Copper Zinc - superoxide dismutase; SP, substance p; TH, tyrosine hydroxylase; (↑) increase; (↓) decrease; NC, no changes.

Evidence of the neuroprotective effect of CBD may be found in a hemiparkinsonian model 6-OHDA injury. However, neurorestorative effects were not evident when CBD was administered one week after injury with 6-OHDA (García-Arencibia et al., 2007). The joint administration of intraperitoneal CBD and intrastriatal 6-OHDA showed increased levels in the mRNA of the enzyme superoxide dismutase Cu/Zn (SOD Cu/Zn) (García-Arencibia et al., 2007), which suggests that CBD affects the expression of antioxidant enzymes, an effect which may, thus, decrease ROS levels in the striatum (Indo et al., 2015). In addition to the probable antioxidant role of CBD, an anti-inflammatory role was demonstrated when a marijuana extract enriched with CBD was administered to rats injured with 6-OHDA i.c.v. The dose was adjusted to an approximate concentration of 3 mg/kg and administered intraperitoneally. It was found that the decrease observed in the levels of OX-42, a marker of reactive microglia, may have induced an increase in the dopaminergic phenotype, as observed via TH immunohistochemistry conducted (García et al., 2011).

Other models of PD enable the evaluation of the effect of neuroprotective drugs. The neurotoxin reserpine decreases dopamine concentrations, leaving dopaminergic synaptic vesicles without a neurotransmitter, thus producing reversible parkinsonism. In an animal PD model, two 1 mg/kg reserpine doses were administered for six days along whit a CBD 0.5 and 5 mg for seven days, resulting in a decrease in catalepsy behavior, a decrease in vacuous chewing movements, and an attenuation of reserpine-induced memory deficit (Peres et al., 2016). Another study found neither neuroprotective effect of CBD in 1-Methyl-4-phenyl-1,2,3,6-tetrahydropyridine (MPTP) models in mice, nor beneficial effects on motor behavior or haloperidol-induced catalepsy (Celorrio et al., 2017).

Crivelaro do Nascimento et al. (2020) observed the neuroprotective effect of CBD in parkinsonian models in order to evaluate non-motor behaviors. After administrating 6-OHDA in the dorsolateral striatum for 21 days, they induced nosciceptive behaviors via the tail flick, hot plate, von frey, and acetone drop test, cannabidiol caused antinosciceptive effects at intraperioteneally administered doses of 10 and 1,000 mg/kg (Crivelaro do Nascimento et al., 2020). This antinosciceptive effect is likely to be more pronounced on interaction between CBD and the TRPV-1, CB2, and GPR55 receptors due to their analgesic and anti-inflammatory properties (Bisogno et al., 2001; Fernández-Ruiz et al., 2010b).

*In vitro* PD models enable the determination of the effect of neuroprotective drugs on cell viability, the modulatory role of the drug, and the signaling pathway that they active, namely the molecule of interest in the present study (Table 3). The most commonly studied cell culture has been the SH-SY5Y neuroblastoma culture, with one study finding that when the neurotoxic MPP^+^ was administered at concentrations of 1 and 7 mM, phenotype and cell viability decreased. However, when CBD was administered at 10 μM, cell viability increased (Gugliandolo et al., 2020), although no differences were observed on the administration of either a 1 and 2.5 µM CBD concentration (Carroll et al., 2012) and in 6-OHDA models (Schönhofen et al., 2015). In the MPP^+^ model at concentrations of 100 μM, the protective effect of 1 µM CBD concentrations was notable in PC12 pheocromocytoma cells, while increased cell differentiation and an increase in the NGF markers, synaptophysin, synapsin-1, and the GAP-43 protein, proteins that promote neural proliferation, were also observed (Santos et al., 2015). The mTOR pathway and the MAPK pathway, signaling pathways that promote cell survival and decrease the level of cell death markers such as Caspase-3 and Bax, are the signaling pathways that are activated by CBD (Gugliandolo et al., 2020).

**TABLE 3:** Research reports of effect CBD on various *in vitro* models of PD.

Cell culture	Cell toxicity induced	Temporary cell toxicity	CBD treatment	Biochemical markers	Proteins evaluated	Author (Reference)
SH-SY5Y	MPP^+^ [7mM]	48 h	CBD [0.01, 0.1 y 1 µM] Joint administration with MPP^+^	(NC) LDH release	Not evaluated	Carroll et al. (2012)
PC12	MPP^+^ [100 µM and 1 mM]	24 h	CBD [1, 5 y 10 µM] Joint administration with MPP^+^	↑ Cell viability MTT assay		Santos et al. (2015)
		72 h	CBD [1 µM]	↑ Differentiation cellular	↑ NGF (ELISA) ↑ Synaptophysin (WB) ↑ Synapsin I (WB) ↑ GAP-43 (WB)	
		72, 96, 120, 144, 168 h		↑ Differentiation cellular whitout MPP^+^		
SH-SY5Y	Without toxin	Without toxin	CBD [1 µM] 72 h	(NC) Differentiation cellular	Not evaluated	
SH-SY5Y	6-OHDA [6.25 µM]	24 h	CBD [2.5 µM]	(NC) Cell viability MTT assay	Not evaluated	Schönhofen et al. (2015)
SH-SY5Y	MPP^+^ [1 mM]	24 h	CBD [10 µM]	↑ Cell viability MTT assay		Gugliandolo et al. (2020)
		48 h			↓ Caspase-3 (WB) ↓ Bax (WB) ↓ PARP-1 (WB) ↑ TH (WB) ↑ pERK ½/ERK2 (WB) ↑ pAKT/AKT (WB) ↑ pmTOR/mTOR (WB) (NC) Beclin-1 (WB) ↓ LC3-II/LC3-I (WB)	

6-OHDA, 6-hydroxydopamine; Bax, BCL2 associated X; CBD, cannabidiol; ELISA, enzyme-linked immunosorbent assay; GAP-43, growth associated protein 43; LC3, microtubule-associated protein 1A/1B-light chain 3; LDH, lactate dehydrogenase; MPP^+^, 1-methyl-4-phenylpyridinium; MTT, 3-(4,5-Dimethylthiazol-2-yl)-2,5-Diphenyltetrazolium Bromide; NGF, nerve growth factor; pAKT, protein kinase B phosphorylated; PARP-1, poly (ADP-ribose) polymerase-1; pERK, extracellular-signal-regulated kinase phosphorylated; pmTOR, mammalian target of rapamycin phosphorylated; TH, tyrosine hydroxylase; WB, western blot; (↑) increase; (↓) decrease; NC no changes.

## The Role of Cannabidiol in Levodopa-Induced Dyskinesia

The word *dyskinesia* is derived from the Greek word *dis*, meaning difficult or abnormal, and *kinesis*, meaning movement, and is used to indicate abnormal involuntary movements (AIMs) (Rascol et al., 2010). Initially, the administration of L-3,4-dihydroxyphenylalanine (l-DOPA), the precursor of DA, produces significant motor symptom improvements in PD patients, reducing tremor, muscle stiffness, difficulty initiating gait, and bradykinesia (slow movements) (Malek et al., 2019). The beneficial effects of l-DOPA last for approximately five years from the start of treatment, considered a “honeymoon period” between l-DOPA and PD, although they can sometimes last longer. After chronic l-DOPA administration, most patients develop motor fluctuations (*on-off*) or dyskinesias, due to increases and decreases in l-DOPA plasma levels (Cotzias et al., 1969).

Normally starting on the side contralateral to the hemisphere most affected by PD and occurring first in the lower extremities, LIDs are either choreiform or dystonic (Mones et al., 1971; Thanvi et al., 2007; Calabresi and Standaert, 2019). Factors such as the age of PD onset and its severity, gender, and the l-DOPA dose administered are factors related to the onset and intensity of LIDs (Eusebi et al., 2018; Zhou et al., 2019), which commonly affects the extremities, head, neck, and trunk, and are characterized by rapid and irregular movements (Luquin et al., 1992; Thanvi et al., 2007; Rascol et al., 2010).

l-DOPA-induced dyskinesias are classified in terms of the plasma levels of the drug administered and the appearance of certain symptoms post-administration. The symptoms that appear when l-DOPA concentrations are at their highest circulation, known as peak-dose dyskinesias, are characterized by stereotyped, ballistic, or choreiform movements (Luquin et al., 1992; Rascol et al., 2010) are usually the most common, and have the greatest impact on quality of life (Luquin et al., 1992; Pahwa et al., 2019). The dyskinesias that begin to occur when l-DOPA reaches its half-life have been termed diphasic and mainly cause rapidly alternating stereotyped movements in the legs (Luquin et al., 1992), as well as ballistic kicking or dystonia (Rascol et al., 2010; Pandey and Srivanitchapoom, 2017). Given that it occurs between the *on* and *off* phases, this period presents mixed dyskinesias, which occur at a low incidence and are difficult to treat (Luquin et al., 1992; Thanvi et al., 2007; Vijayakumar and Jankovic, 2016). When l-DOPA falls to a low level of circulation, the movements caused are known as *off* dyskinesias, in which patients usually suffer from a dystonic posture, especially in the morning (Marconi et al., 1994), and problematic lower limb sensations (Thanvi et al., 2007; Rascol et al., 2010).

Few studies have obtained evidence of the antidyskinetic activity of CBD, whit its pleiotropic effects (Devinsky et al., 2014) depending on the concentration administered (Jones et al., 2010). As determining the molecular mechanism that is involved in LIDs (see below) is a complex process, the preclinical and clinical evidence depends on the experimental design used to evaluate CBD as a potential treatment for reducing both PD symptoms and the adverse effects of l-DOPA administration. A study was conducted on tardive dyskinesias, which are related to the *nigro-striatal* pathway, which, itself, is affected by the administration of reserpine (1 mg/kg), a drug which has been shown to reduce glutamate consumption (Burger et al., 2005). Said research obtained favorable results in behavioral evaluations via the administration of CBD (0.5 and 5 mg/kg), finding improved memory and reductions in oral dyskinesia and the cataleptic effect (Peres et al., 2016), which describes the subject’s inability to correct an imposed posture (Pertwee, 1972), without modifying locomotor activity and anxiety in the model (Peres et al., 2016).

As previously mentioned, given that l-DOPA must be metabolized in the serotonergic neurons, the participation of these neurons in LIDs is an important factor to consider. It has been shown that the pharmacological silencing of serotonergic neurons can be accomplished via the agonists of serotonergic auto-receptors. Therefore, several studies show a decrease in LIDs as induced by selective agonists of the 5-HT1 receptors in animal models of PD (Bibbiani et al., 2001; Muñoz et al., 2008). As CBD has been shown to interact with the 5-HT1A receptor, this phytocannabinoid is able to modulate serotonergic neurotransmission (Magen et al., 2010; Zanelati et al., 2010; Espejo-Porras et al., 2013). *In vitro* studies have demonstrated that, at concentrations higher than 10 mM, CBD is able to activate 5-HT1A; however, at concentrations of 100 nM, it is also able to improve the agonist capacity of the 5-HT1A receptor in rat brainstem membranes (Rock et al., 2012). Given this effect of CBD on serotonergic receptors, it has been proposed as an anxiolytic (Gomes et al., 2011) and antidepressant agent (Zanelati et al., 2010). Moreover, it has been shown, at high doses (>10 mg/kg), to modify motor behavior, wherein 20 mg/kg doses administered during motor behavior were not antagonized by rimonabant, a CB1 antagonist, but were antagonized by a selective 5-HT1A receptor antagonist (WAY100,635; 0.5 mg/kg) (Espejo-Porras et al., 2013). These data demonstrate that CBD has significant effects on both motor behavior and the 5-HT1A receptor. While it is likely that CBD exerts effects on serotonergic receptors during LIDs, the ability of CBD to activate various mechanisms that exert a synergistic effect on PD and LIDs should not be excluded.

The antidyskinetic effect of CB1 activation, by means of AEA or WIN55212–2, is exhibited only when co-administered with a TRPV1 receptor antagonist (Morgese et al., 2007; Martinez et al., 2012; Martinez et al., 2015; Dos Santos-Pereira et al., 2016), namely capsazepine (CPZ) or N-arachidonoyl serotonin (AA-5-HT) (Dos Santos-Pereira et al., 2016). In addition, AA-5-HT inhibits the FAAH (Gobira et al., 2017) mechanism that increases concentrations of AEA, an ECB with a greater affinity with the CB1 receptor (Lam et al., 2005). However, the use of the TRPV1 antagonist (CPZ) is necessary in order to demonstrate the antidyskinetic effects of the inhibition of FAAH. Promising results were obtained in research conducted on the 6-OHDA model in mice with a dorsolateral striatum lesion. After l-DOPA treatment for 21 days (50 mg/kg/day, ip), CBD and capsazepine (CPZ, 1 and 5 mg/kg, ip, respectively), an antagonist of TRPV1, were administered during the last days of an AIMs evaluation, showing a decrease in AIMs with the administration of CBD + CPZ (Dos Santos-Pereira et al., 2016). These results were similar to those obtained with the use of N-araquidonoil serotonin (AA-5-HT), an eCB antagonist of FAAH and TRPV1 (Gobira et al., 2017). While these effects were previously evaluated using synthetic cannabinoids, with similar results obtained, it should be noted that the administration of WIN55212–2 (0.5 and 1 mg/kg ip) was shown to have antidyskinetic effects. Although the study’s authors used WIN55212–2 as a selective agonist for CB1 (Martinez et al., 2012), it is known to have both a higher affinity for CB2 and the ability to inhibit TRPV1 (Morgese et al., 2007).

**FIGURE 6:**
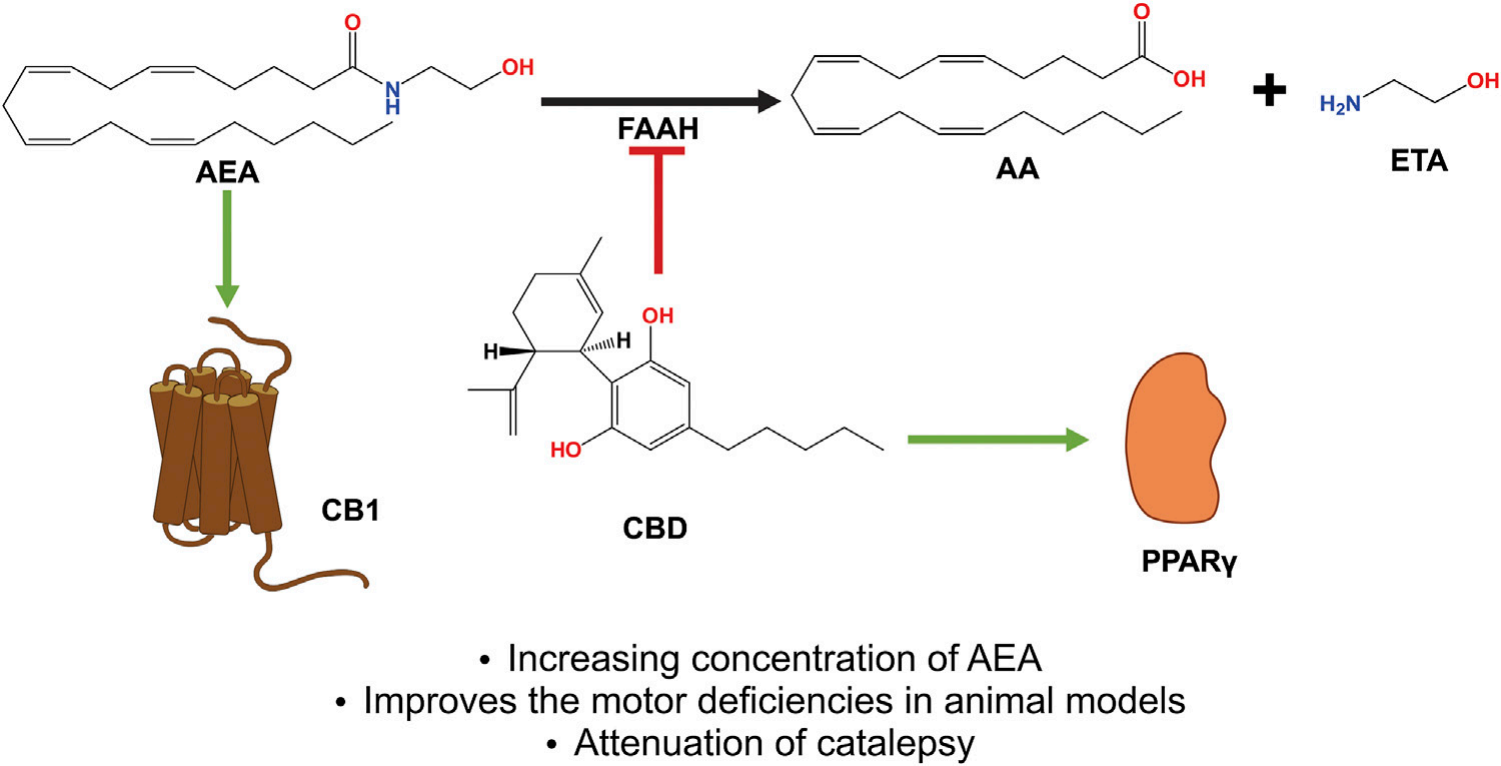
CBD acts as an indirect agonist of the CB1 receptor by inhibiting the enzyme that degrades AEA and FAAH, thus increasing the concentration of said eCB, which has a greater affinity to activating CB1, thus exerting neuroprotective and antiparkinsonian effects. In addition, CBD activates the PPARγ receptors, which have been shown to be involved in neuroinflammatory processes. Abbreviations: CBD, cannabidiol; CB1, cannabinoid receptor type 1; AEA, anandamide; FAAH, fatty acid amide hydrolase; eCB, endocannabinoids; PPARγ, peroxisome proliferator activated receptor γ.

The mechanism by which AIMs are reduced by CBD + CPZ suggests that improving endocannabinoid tone and blocking TRPV1 receptors exerts a compensatory effect on aberrant endocannabinoid transmission, which occurs in the striatum of dyskinetic rats in 6-OHDA models after l-DOPA treatment (Wang et al., 2018). The increase in AEA, which preferentially activates CB1 over TRPV1 (McPartland et al., 2007; Lam et al., 2005), is the result of the inhibition of FAAH by CBD, which can also activate TRPV1 (Peres et al., 2018) (Figure 6). While stimulating CB1 alone reduces the hyperactivity of the cAMP/PKA pathway, which has been implicated in the development of dyskinesias (Aubert et al., 2005; Martinez et al., 2012), the coactivation of CB1 and TRPV1 generates the opposite effect (Lam et al., 2005), with their participation in the induction of LTP also observed (Cui et al., 2018). This explains why CBD or FAAH antagonist (URB597) treatments do not reduce AIMs; however, when they are co-administered with CPZ, they exert an antidyskinetic effect on all types of AIMs (Morgese et al., 2007; Martinez et al., 2015; Dos-Santos-Pereira et al., 2016).

An examination of the above-described mechanisms reveals evidence concurring with the results obtained via the subchronic administration of CBD + CPZ, which reduces the severity of LIDs and reduces the levels of biochemical markers such as pERK, pAcH3, NF-κβ, and COX-2 (Dos-Santos-Pereira et al., 2016), which increase with chronic l-DOPA treatment (Santini et al., 2007; Bastide et al., 2014). Both pERK and pAcH3 are generated by D1-receptor sensitization (Park et al., 2014), which maintains an overactivated signaling pathway and which, in turn, results in an increase in the level of these markers (Aubert et al., 2005). NF-κβ and COX-2 are characteristic of a neuroinflammatory process, with their levels increasing due to the depletion of DA and the neurotoxicity caused by l-DOPA (Bortolanza et al., 2015; Pisanu et al., 2018), while a reduction in their levels causes the activation of PPARγ (Randy and Guoying, 2007) and occurs due to CBD’s own anti-inflammatory effects (Peres et al., 2018).

**FIGURE 7:**
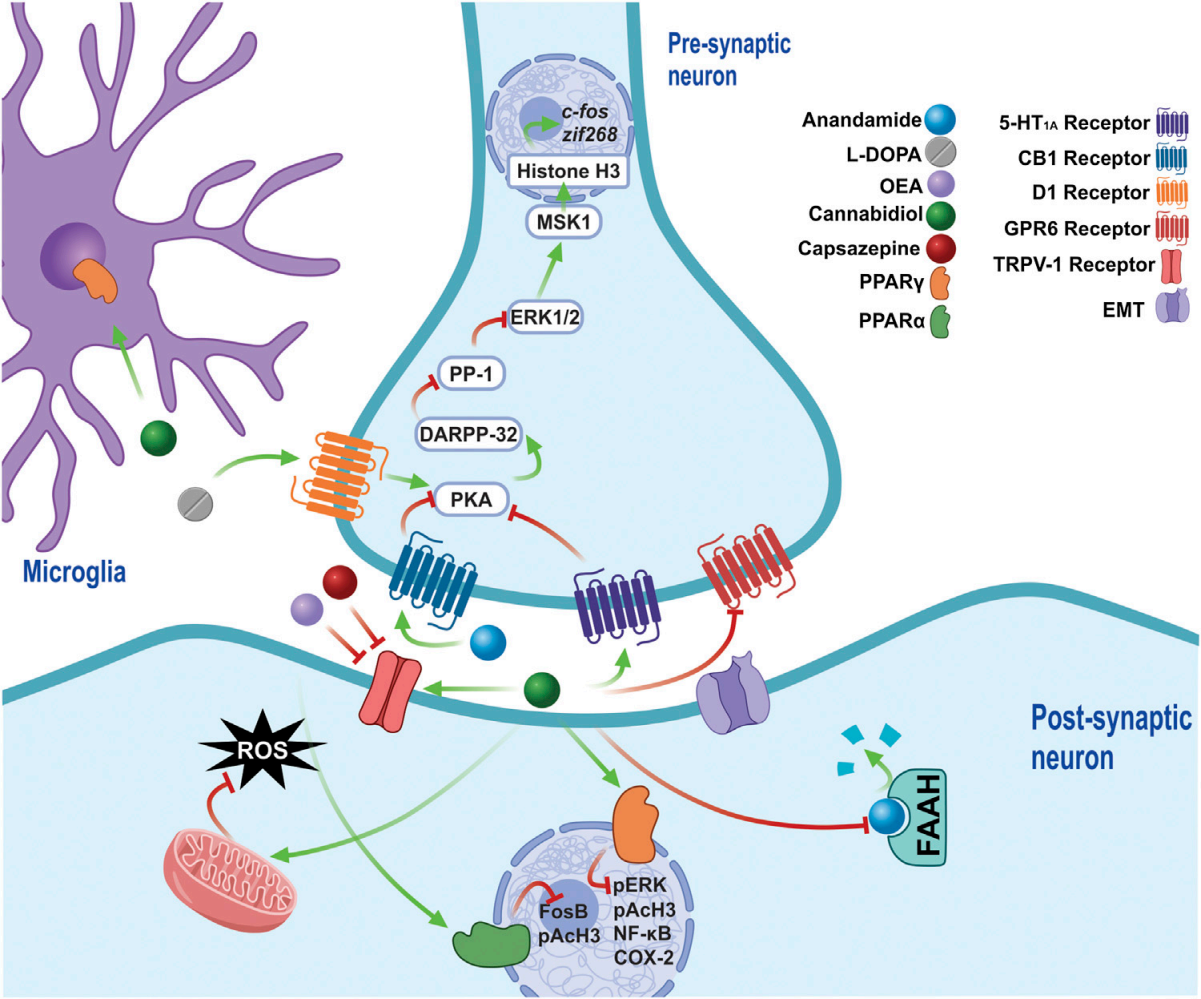
The chronic administration of l-DOPA leads to a sensitization of D1 receptors, which maintain the over-activation of PKA in LIDs. PKA regulates the pathway that activates DARPP-32, which inhibits the modification by PP-1, of ERK1/2 signaling, which acts on nuclear targets, such as MSK1, and, along with histone H3, regulates the expression of early genes such as c-fos and zif268. CBD exerts antidyskinetic effects by increasing AEA concentration by inhibiting of FAAH, thus stimulating the CB1 receptors, which decrease PKA activity. The CB1 requiere the co-administration of a TRPV1 inhibitor (CPZ), because they stimulate TRPV1 via AEA and CBD, both of which generate opposite effects to the activation of CB1. Furthermore, increased OEA is generated via the inhibition of FAAH, an endocannabinoid able to block TRPV1 and stimulate PPARσ receptors, reducing biochemical markers such as FosB and pAcH3. In addition, CBD activate the 5-HT1A receptor, a receptor that had previously only been implicated in the anticataleptic effect of CBD. By activating PPARγ receptors, CBD reduces the levels of molecular markers involved in LIDs, such as pERK, pAcH3, NF-Kβ and COX-2, while it also generates an anti-inflammatory effect by stimulating said receptors, which are present in the glia. Furthermore, CBD is able to reduce oxidative damage, decreasing the production of ROS by increasing the activity of mitochondrial complexes. The inverse agonism that CBD exerts on GPR6 could form part of its antidyskinetic mechanisms. Abbreviations: l-DOPA, L-3,4-Dihydroxyphenylalanine; D1, Dopamine receptor 1; PKA, cAMP-dependent protein kinase; LID, l-DOPA-induced dyskinesias; DARPP-32–32 KDa Phosphoprotein regulated by cAMP and dopamine; PP-1, phosphoprotein 1; ERK, extracellular signal-regulated kinase; MSK-1, mitogen and stress regulated protein kinase; CBD, cannabidiol; AEA, anandamide; FAAH, fatty acid amide hydrolase; TRPV1, transient potential receptor V1; CPZ, cpazazepine; OEA, oleoylethanolamide; PPARα, peroxisome proliferator activated receptor α; pAcH3, Histone 3 phosphoacetylation; 5HT1A, Serotonin receptor 1A; PPARγ, peroxisome proliferator activated receptor γ; NF-Kβ, nuclear factor Kβ; COX-2, cyclooxygenase 2.

It has been shown that the administration of the eCB oleoylethanolamide (OEA) generates antidyskinetic effects (González-Aparicio and Moratalla, 2014), results which show that its mechanisms block TRPV1 and stimulate PPARσ (Almási et al., 2008; Thabuis et al., 2008). Moreover, a decrease in the levels of markers such as FosB and pAcH3 has also been observed (González-Aparicio and Moratalla, 2014). In addition, an increase in the concentration of OEA has been reported with the administration of URB597 in an EP model injured with MPTP (Celorrio et al., 2016), as it shares the same degradation pathway as AEA (Figure 7) (Thabuis et al., 2008), with the same study also observing a motor deficit improvement (Celorrio et al., 2016).

Other molecular targets of CBD, such as the GPR6 orphan receptor, which is mainly expressed in the *striato-pallidal* neurons of the striatum, have been studied in recent years (Lobo et al., 2007). One study found an AIMs reduction in knockout mice after GPR6 ablation, as well as a cAMP reduction and an increase in both DA levels and the phosphorylation of DARPP-32 in the striatum. This suggests that blocking GPR6 exerts an antidyskinetic effect (Oeckl et al., 2014), with research showing that CBD acts as an inverse agonist on GPR6 (Laun and Song, 2017), an effect forming part of its therapeutic mechanisms.

Clinical evaluations of CBD have found improvements in terms of certain symptoms, depending on the doses administered (Jones et al., 2010). In an open preliminary pilot study, which evaluated dystonia under weekly CBD dose escalations (100 mg/week), one of the patients, who was received 1,000 mg/day l-DOPA doses, presented a 50% improvement (Consroe et al., 1986). Given that it is a type of LID (Marconi et al., 1994), the use of CBD, at appropriate doses, is suggested to reduce the severity of dystonia. An increase in hypokinesia has been reported with the administration of high CBD doses (300–400 mg/day); however, there is currently no complete understanding of its pharmacokinetics, although significant improvements in symptoms have been observed in a dosage range of 1–50 mg/kg/day in different pathologies (Millar et al., 2019). Furthermore, no changes were found when quantifying brain-derived neurotrophic factor (BDNF), the level of which decreases in PD, while a greater susceptibility to LID was found in patients with a polymorphism in the gene encoding BNDF (Howells et al., 2000; Foltynie et al., 2009). Both the increase in BDNF (Sales et al., 2019) and the improvement in LID symptoms that can be caused by CBD require further study in order to be applied in clinical practice. Moreover, it must also be established whether results improve with the use of other phytocannabinoids that exert a synergistic effect (Samarut et al., 2019).

## Conclusion

The bibliographic evidence shown in the present review suggests the clinical utility of CBD for treating both LIDs and the motor symptoms of PD, as well as the neuromodulatory, neuroprotective and antidyskinetic effects of CBD in animal models and *p*D. Furthermore, the evidence shown on the pharmacological mechanisms and molecular interactions of CBD with various receptors may explain the wide range of therapeutic utility in various neurological disorders.

Despite the promising results for CBD pharmacology, unknowns remain about dosages and mechanisms of action. However, the essential role of CBD as an antioxidant and anti-inflammatory is affirmed, as these processes are important in the pathogenesis of *p*D. The neuromodulatory mechanism of CBD in the BG circuit remains to be studied in greater depth, in order to establish this phytocannabinoid’s physiological role and function as a coadjuvant in PD.

## Author Contributions

FP, AM-A, AP-M, and IL performed the bibliography searches and participated in the manuscript writing, all authors reviewed and approved the final version of the manuscript. FP and AM-A made all the figures in this review.

## Funding

This study was supported by grants from VIEP-BUAP 2019-2020, awarded to IL, while FP-received support via a scholarship from CONACYT-Mexico (732793).

## Conflict of Interest

The authors declare that the research was conducted in the absence of any commercial or financial relationships that could be construed as a potential conflict of interest.

## Abbreviations

2-AG 2-arachidonoyl-glycerol; 6-OHDA 6-hydroxydopamine; AEA, anandamide; BG, basal ganglia; CB1, cannabinoid receptor type-1; CB2, cannabinoid receptor type-2; CBD, cannabidiol; CBDA, cannabidiol acid; CBRs, cannabinoid receptors; CPu, caudate-putamen; eCBs, endocannabinoids; ECS, endocannabinoid system; FAAH, fatty acid amide hydrolase; GPe, globus pallidus external; GPi, globus pallidus internal; GPR55, G protein receptor-55; l-DOPA Levodopa; LID l-DOPA-induced dyskinesia; MPTP 1-Methyl-4-phenyl-1,2,3,6-tetrahydropyridine; PD, Parkinson’s disease; PPAR-γ, Peroxisome proliferator-activated gamma; SNpc, substantia nigra pars compacta; SNpr, substantia nigra pars reticulata; STN, subthalamic nucleus; THC Δ9-tetrahydrocannabinol; THCA Δ9-tetrahydrocannabinol acid; TRPV-1, transient receptor potential vanilloid-1.
